# Brucellosis in Infant after Familial Outbreak

**DOI:** 10.3201/eid1408.080325

**Published:** 2008-08

**Authors:** Alexandros C. Makis, Georgios Pappas, Emmanouel Galanakis, Nikolaos Haliasos, Antigoni Siamopoulou

**Affiliations:** * University of Ioannina, Ioannina, Greece; † Institute of Continuing Medical Education of Ioannina, Ioannina; ‡ University of Crete, Heraklion, Greece

**Keywords:** brucellosis, transmission, infantile, Greece, letter

**To the Editor:** Brucellosis is a known cause of small household outbreaks (*1*,*2*), usually attributed to exposure of all infected family members to the animal/animal product pathogen source. Although the means of disease transmission is well delineated (*3*), in certain cases the pathogen’s entry into the human body cannot be clearly defined; this has led to suggestions of direct human-to-human transmission and also to the increasing recognition of airborne brucellosis, which is important in the context of the role of *Brucella* spp. as potential biological weapons (*4*). Another understudied transmission route is entry by direct contact through skin and mucosal abrasions. We report a case of infantile brucellosis in which airborne transmission in the context of familial brucellosis or indirect contact with the animal source through other family members was considered the only possible means of infant infection.

In 2006, a 2.5-month-old girl was admitted to the Pediatric Department of the University Hospital of Ioannina, in a region of northwestern Greece where animal and human infection from *Brucella melitensis* is still common (*5*,*6*). She had a 2-week history of poor feeding and a 5-day history of swelling of the right wrist. She was born after 38 weeks’ gestation with a birthweight of 3,050 g and was fed formula milk exclusively. Results of the physical examination were normal except the finding of a tender swelling of the right wrist. The infant came from a family of shepherds, and her father and paternal grandfather had been treated for brucellosis 10 and 22 months ago, respectively. At that time, the whole family was screened for additional cases; screening was also often repeated at the patients’ followup examinations. On admission, laboratory tests showed characteristic relative lymphocytosis (leukocytes 10.5 × 10^9^/L, 65.3% lymphocytes) and an increase in inflammatory markers (C-reactive protein, 22 mg/L, and erythrocyte sedimentation rate, 47 mm/h). Results of a wrist x-ray were normal. Because brucellosis was suspected, serum agglutination test, ELISA, and blood PCR for *B. melitensis* were performed. Agglutination titer was 640; ELISA immunoglobulin M (IgM) antibodies and PCR results were positive. No organisms were grown in blood culture.

All family members were rescreened. The father and paternal grandfather had negative serum agglutination and ELISA IgM and positive IgG serologic results, indicating past infection. The mother and paternal grandmother were again negative. Veterinary investigation showed active disease in a few sheep of the family’s herd. The infant was treated with a combination of oral trimethoprim-sulfamethoxazole and rifampin for 6 weeks. The course of the illness was uneventful, and she recovered completely. Followup PCR results were negative for *B. melitensis.* Six months later, only an ELISA IgG had positive results; IgM and IgA antibody and agglutination test results were negative. The patient remains without relapse 2 years after treatment.

Awareness of brucellosis is low in disease-endemic areas, including knowledge of its transmission potential and its medical consequences.. As a consequence, familial clusters of brucellosis are the norm. Recognition of a human case should prompt investigation of other family members so that early recognition and treatment for other household case-patients are possible. However, limitations in eradicating the initial animal disease source may lead to continuous exposure and appearance of new cases after a protracted period, or to infection of new household members.

Our case raises the need for awareness of the transmission dynamics of *Brucella* spp. because the disease emerged in a household member who did not have any direct contact with the animal source or any related products. The baby was not breastfed and had not digested raw milk. Her feeding bottle was specifically used for formulated milk and for feeding her only. Ingestion of breast milk from an infected mother (*7*) and vertical transmission transplacentally or during delivery are acknowledged means of transmission (*8*), but in this case the mother had never had brucellosis (she had been repeatedly screened during her husband’s initial disease and followup). Neither previously infected household member had any clinical or laboratory sign of relapse or residual disease. The infant was never in contact with the infected animals and never carried to the sites where they were housed or taken out for grazing. Thus, the only way for the infant patient to be infected would be environmental exposure. Since the infant had not been carried to the animal sites, the pathogen must have been transmitted to the household by the clothes or skin of the father or grandfather, who had been shepherding the infected animals. Subsequently, the baby became infected either by inhaling infected aerosolized particles or by direct transmission of the pathogen through minor skin abrasions (which were specifically looked for on admission but were not seen) or mucosal surfaces.

This case suggests that *B. melitensis* may even affect persons who are not directly exposed to infected animals, through direct contact with contaminated persons or the environment. In this context, brucellosis can be considered as not simply a household disease but as a disease of the house.
